# Continuous long-range measurement of tonic dopamine with advanced FSCV for pharmacodynamic analysis of levodopa-induced dyskinesia in Parkinson’s disease

**DOI:** 10.3389/fbioe.2024.1335474

**Published:** 2024-01-24

**Authors:** Jeongrak Park, Seongtak Kang, Yaebin Lee, Ji-Woong Choi, Yong-Seok Oh

**Affiliations:** ^1^ Department of Brain Science, Daegu Gyeongbuk Institute of Science and Technology (DGIST), Daegu, Republic of Korea; ^2^ Department of Electrical Engineering and Computer Science, Daegu Gyeongbuk Institute of Science and Technology, Daegu, Republic of Korea

**Keywords:** Parkinson’s disease, levodopa-induced dyskinesia, dopamine, fast-scan cyclic voltammetry, levodopa pharmacodynamics

## Abstract

Levodopa, a dopamine prodrug, alleviates the motor symptoms of Parkinson’s disease (PD), but its chronic use gives rise to levodopa-induced dyskinesia (LID). However, it remains unclear whether levodopa pharmacodynamics is altered during the progressive onset of LID. Using *in vivo* fast-scan cyclic voltammetry and second-derivative-based background drift removal, we continuously measured tonic dopamine levels using high temporal resolution recording over 1-h. Increases to tonic dopamine levels following acute levodopa administration were slow and marginal within the naïve PD model. However, these levels increased faster and higher in the LID model. Furthermore, we identified a strong positive correlation of dyskinetic behavior with the rate of dopamine increase, but much less with its cumulative level, at each time point. Here, we identified the altered signature of striatal DA dynamics underlying LID in PD using an advanced FSCV technique that demonstrates the long-range dynamics of tonic dopamine following drug administration.

## 1 Introduction

Parkinson’s disease (PD) is a neurodegenerative disease that causes motor defect symptoms, such as tremors, rigid posture, and slow movement (Port et al., 2021). PD is characterized by severe dopaminergic neuron loss within the substantia nigra pars compacta (SNpc) (Ma et al., 1997; Przedborski, 2005). Patients with PD frequently use medication to alleviate PD-related motor defect symptoms. Levodopa, a dopamine (DA) precursor, is the most common medication used for treating PD and functions by elevating tonic DA levels to manage the hypodopaminergic condition. However, long-term use of this medication can lead to the development of levodopa-induced dyskinesia (LID) (Friedman, 1985) and abnormal involuntary movements in arms, legs, body, and face. The precise cause of dyskinesia following levodopa usage remains unclear. However, one prominent theory is that pulsatile levodopa intake leads to dopaminergic stress on medium spiny neurons (MSNs). This stress induces MSNs to alter DA receptor (DAR) expression and sensitivity (Gerfen, 2003; Aubert et al., 2005; Girasole et al., 2018), which in turn causes a strength imbalance in direct and indirect motor pathways. Although this postsynaptic mechanism highlights abnormal changes to MSNs and their downstream pathways (Parker et al., 2018; Fabbrini and Guerra, 2021; Kaasinen et al., 2021), it is unclear whether levodopa pharmacodynamics are altered with the progression of dyskinesia.

DA signaling can be divided into phasic and tonic signals. Phasic DA levels describe fast DA changes over a brief period (e.g., within a sub-minute window). In contrast, tonic DA levels describe slower DA changes over extended periods (e.g., minutes to hours) (Berke, 2018). Several *in vivo* DA sensing techniques are used to monitor DA dynamics in the field (Labouesse et al., 2020; Ravariu, 2023). Among these techniques, microdialysis, in combination with analytical high-performance liquid chromatography (HPLC), is used to measure tonic DA levels with a high degree of sensitivity and selectivity for DA. Microdialysis often uses a sampling period longer than several minutes, limiting this measurement’s temporal resolution. Furthermore, *in vivo* implantation of a thick microdialysis probe (240–770 μm outer diameter) often causes severe tissue damage and neuroinflammation, which limits the accurate measurement of physiological DA dynamics (Clapp-Lilly et al., 1999). On the other hand, G protein-coupled receptor (GPCR) DA biosensors such as dLight (Patriarchi et al., 2018) and GRAB_DA_ (Sun et al., 2020), combined with fiber photometry, are well-suited to measure phasic DA release with high sensitivity and spatiotemporal resolution. However, following continuous exposure to an excitation light, fluorophore photobleaching may hamper its ability to monitor tonic DA levels long-term. Furthermore, the narrow detectable dose range of DA activity is unfavorable to characterizing huge fluctuations of tonic DA following acute levodopa administration (Park et al., 2010; Wakabayashi et al., 2016). Another dopamine sensing techniques, such as Field-effect transistor (FET) (Liu et al., 2021), and organic electrochemical transistor (OECT) (Xie et al., 2020) were emerging to increase sensitivity and selectivity for a dopamine. But these techniques yet to be verified *in vivo* long-term usage with stability and biocompatibility. Conversely, fast-scan cyclic voltammetry (FSCV) enables high temporal resolution and sensitivity in measuring phasic levels primarily in the short term (Garris et al., 1997; Park et al., 2010; Schluter et al., 2014; Wakabayashi et al., 2016). However, the background charging current on an implanted carbon fiber microelectrode (CFME) gradually increases over the entire recording session, interfering with continuous tonic DA level tracking over a 1-min window (Venton and Cao, 2020). Several attempts to use FSCV have compromised the temporal resolution for long-term estimates of tonic DA levels (Atcherley et al., 2015; Oh et al., 2016; Oh et al., 2018; Taylor et al., 2019). Recently, we introduced the advanced FSCV technique with second-derivative-based background drift removal (SDBR), which identifies the continuous dynamics of tonic DA levels with the high temporal resolution offered by standard FSCV data recorded over long durations. This dopamine sensing platform has been validated on its sensitivity and selectivity by *in vitro* and *in vivo* experiments (Kang et al., 2022).

Here, we applied an advanced FSCV system to trace tonic DA levels following acute levodopa administration. We found that chronic drug administration in PD accelerates excessive hyperdopaminergic conditions correlated with the onset of dyskinetic behavior. Due to the capacity of the FSCV system to reflect accurate levodopa pharmacodynamics, we identified the altered signature of tonic DA dynamics underlying LID in the striatum of the PD model.

## 2 Materials and methods

### 2.1 Animals

Adult male C57BL/6J mice (body weight: 30–35 g) purchased from Orient Bio (South Korea) were used for these *in vivo* experiments. Animals were housed in 3-5 per cage on a 12-h light/dark cycle with *ad libitum* free access to food and water. All experiments were performed during the light cycle phase with the same procedures, and the data, behavioral and FSCV measurements, were analyzed with identical parameters. All measured data were used for analysis except dead mice along the 2 weeks of the test period. All animal care and experimental procedures were approved by the Institutional Animal Care and Use Committees (IACUC) at Daegu Gyeongbuk Institute of Science and Technology (DGIST-IACUC-20073002–0005) and were performed by experts by approved procedures.

### 2.2 Stereotaxic surgeries

To generate the 6-OHDA-induced hemi-PD mouse model, the mice were intraperitoneally injected with 5 mg/kg pargyline hydrochloride (abcam, #ab141265) and 25 mg/kg desipramine hydrochloride (Sigma-Aldrich, #D3900) solution 30 min before surgery to increase the selectivity and efficacy of 6-OHDA lesions. Primary anesthesia was induced by adding 5% isoflurane to the chamber, and approximately 2% isoflurane was administered to anesthetized mice using a stereotaxic instrument. Three micrograms of 6-OHDA (Sigma-Aldrich, #H-116) were delivered to the left medial fiber bundle (MFB) at the following coordinates from the bregma: anterior-posterior (A/P) = −1.20; medio-lateral (M/L) = −1.10; and dorso-ventral (D/V) = −5.00. Two weeks after post-operative care, a FSCV carbon probe was implanted into the left caudate putamen (CPu), which is a DA-depleted region in the hemi-PD model at the following coordinates from the bregma: A/*p* = +1.00; M/L = −1.50; and D/V = −2.50 in the hemi-PD model and wild-type mice.

### 2.3 FSCV measurement of tonic DA changes

To conduct DA measurements using the FSCV technique, mice were anesthetized in the 5% isoflurane-filled chamber and fitted with a nose mask. Due to the long measurement duration of over 1 hour, the isoflurane concentration was slowly decreased to 1.5% in mice for the safety of anesthesia. Mouse body temperature was maintained at 37°C using a hot pack. CFME and Ag/AgCl reference electrodes (Pinnacle Technology Inc., Lawrence, KS) were used to measure FSCV within mouse brains. A standard triangular waveform (−0.4–1.3 V) with a scanning rate of 400 V/s was used to capture voltammetric scans. The FSCV waveform was applied for approximately 30 min to stabilize the signal. The sampling rate was 10 Hz. We measured tonic DA level changes for 2 h and analyzed the 70-min window that spanned the time point for drug IP injection. Data acquisition was performed using High-Definition Cyclic Voltammetry software (HDCV, University of North Carolina at Chapel Hill) in conjunction with a WaveNeuro FSCV system (Pine Research Instrumentation, Durham, NC). To extract dopamine signal using SDBR technique from FSCV data, we modeled a background-subtracted voltammogram at specific scan times (t) and voltages (V) around the dopamine oxidation peak voltage. (1)

$${Voltammogram}_{BS}\left(V,t\right)=e^{-\frac{{\left(V-peak\;voltage\right)}^{2}}{2}}{Concentration}_{DA}+Charging\;current\left(t\right),$$ (1a)

If we observe the dopamine peak current after the second derivative of the modeled voltammogram by setting V to peak voltage : (2)

$$\frac{-d^{2}}{dV^{2}}{Voltammogram}_{BS}\left(peak\;voltage,t\right)={Concentration}_{DA}.$$ (2a)

This second derivative, tonic dopamine level can be obtained irrespective of the time-varying charging current level. The dopamine oxidation peak voltage of each sensor was defined as the voltage which has a maximum SDBR value in the range of 0.4–0.7 V in the *in vitro* test. The recorded *in vivo* SDBR value was converted into an estimated DA concentration with linear regression performed using an *in vitro* test result. Detailed procedures and outcomes across all *in vitro* experiments, including sensitivity and selectivity tests, were described in the previous research (Kang et al., 2022). In short, CFME and Ag/AgCl reference electrodes were placed in a beaker filled with 0.05 M, pH 7.4 phosphate-buffered saline (PBS). A single drop of DA was delivered into PBS and mixed with 2 min of stirring. All *in vitro* tests were processed in a Faraday cage for stable FSCV measurement.

### 2.4 Slope calculation of tonic DA level changes

To calculate the slope of DA level changes, we identified two reference coordinates (( $x_{i-d}$ , $y_{i-d}$ ), ( $x_{i}$ , $y_{i}$ )) with a distance of d for a gradient calculation at each data point ( i ). The two reference coordinates were selected as the average value of n+1 samples remove noise from the signal following Eq. 1b:

$$x_{i}=\sum_{i-\frac{n}{2}}^{i+\frac{n}{2}}x_{i}/\left(n+1\right),y_{i}=\sum_{i-\frac{n}{2}}^{i+\frac{n}{2}}y_{i}/\left(n+1\right),$$ (1b)

where an integer n is an even value.

The slope of the i ^th^ sample can be written as

$${slope}_{i}=\frac{y_{i}-y_{i-d}}{x_{i}-x_{i-d}}.$$ (2b)

n = 100 (10 s) and d = 600 (1 min) were used to align the temporal information of both slope and rotations per minute (RPM) to analyze this relationship. We divided the rotational behavior into two states using the maximum rotational RPM peak following levodopa injection. All scatter plots were visually examined using different colors before (black circles) and after (blue, yellow, and orange circles in each week) the maximum peak of the rotational behavior.

### 2.5 Chronic levodopa administration

One day after FSCV carbon probe implantation, the wild-type and hemi-PD mice were delivered 6 mg/kg 3,4-Dihydroxy-L-phenylalanine (Sigma-Aldrich, #D9628), also known as levodopa and 12 mg/kg benserazide hydrochloride (Santa Cruz, #sc-200723) daily for 14 days. Benserazide hydrochloride was used to prevent levodopa from changing DA in peripheral tissues. The vehicle group was administered 6 mg/kg levodopa and 12 mg/kg benserazide hydrochloride following the FSCV recording and behavioral rotation test.

### 2.6 Rotation test

Behavioral measurement was performed in a cylinder (20 cm diameter, 30 cm height) and recorded from above. This included 10 min of habituation and 20 min of recording before pharmacological treatment. After capturing the basal behavioral recording, the mice were delivered either the vehicle or 6 mg/kg levodopa. After treatment, we caught 50 min of recording. Behavioral videos were analyzed using EthoVision XT (Noldus) with 3-body detection points (nose, body, and tail). Behavior patterns were quantified by counting the number of rotations per minute based on a line that connected the upper part of the body to the endpoint of the body. To plot the number of clockwise rotations per minute, we subtracted counterclockwise rotations from clockwise rotations.

### 2.7 Histology

Two weeks after levodopa treatment, the mice were administered either the vehicle or levodopa. Thirty minutes after treatment, the mice were anesthetized and euthanized with 250 mg/kg 2, 2, 2-Tribromoethanol (Sigma-Aldrich, #T48402) and perfused with cold saline and 4% paraformaldehyde solution. The brain was extracted and incubated overnight in a 4% paraformaldehyde solution at 4°C. The following day, the brain was transferred to a 15% and 30% sucrose solution at 4°C for infiltration until the brain was submerged. Brains were frozen in an OCT medium, and 50 µm sectioning was performed using a cryostat (Leica, CM3050S). To perform immunohistochemistry, brain sections were incubated in a blocking buffer (2% goat serum, 0.2% bovine albumin serum, and 0.3% Triton-X 100 in 1X PBS) for 1 h at room temperature. Primary antibodies were diluted using a blocking buffer to generate a primary antibody solution. Tyrosine hydroxylase (abcam, #ab76442) and c-Fos (Cell signaling, #2250S) were applied to the solution. Following 1–3 days of brain section incubation in the primary antibody solution at 4°C, brain sections were washed with a washing buffer (0.2% Triton-X 100 in 1X PBS) for 5 min, and this was repeated three times. The secondary antibody solution was diluted with the washing buffer and included DRAQ5 (ThermoFisher, #62252) for nucleus staining. Goat anti-rabbit Alexa Fluor 488 (ThermoFisher, #A-11008), goat anti-chicken Alexa Fluor 488 (ThermoFisher, #A-11039), and goat anti-rabbit Alexa Fluor 568 (ThermoFisher, #A-11011) were used for the secondary antibody. The secondary antibody staining was performed for 3 h at room temperature. Next, the brain sections were washed with a washing buffer twice for 15 min and 1X PBS once for 15 min. The tissue sections were mounted on glass slides. Each slide was imaged using Nikon A1 confocal microscopy and Carl Zeiss LSM780.

### 2.8 Statistics

All statistical analyses were performed using Prism 7 (GraphPad) and MATLAB R2020b (Mathworks) with appropriate parametric or non-parametric tests. Statistical parameters, including the statistics tests, sample numbers, *post hoc* tests, and statistical significance, are reported in every figure and figure legend. Data were estimated to be statically significant when the *p*-value was under 0.05 by two-way ANOVA, one-way ANOVA, and two-tailed t-tests. All values are shown as mean ± SEM.

## 3 Results

### 3.1 Advanced FSCV technique to trace pharmacodynamics of levodopa in the PD model

**FIGURE 1:**
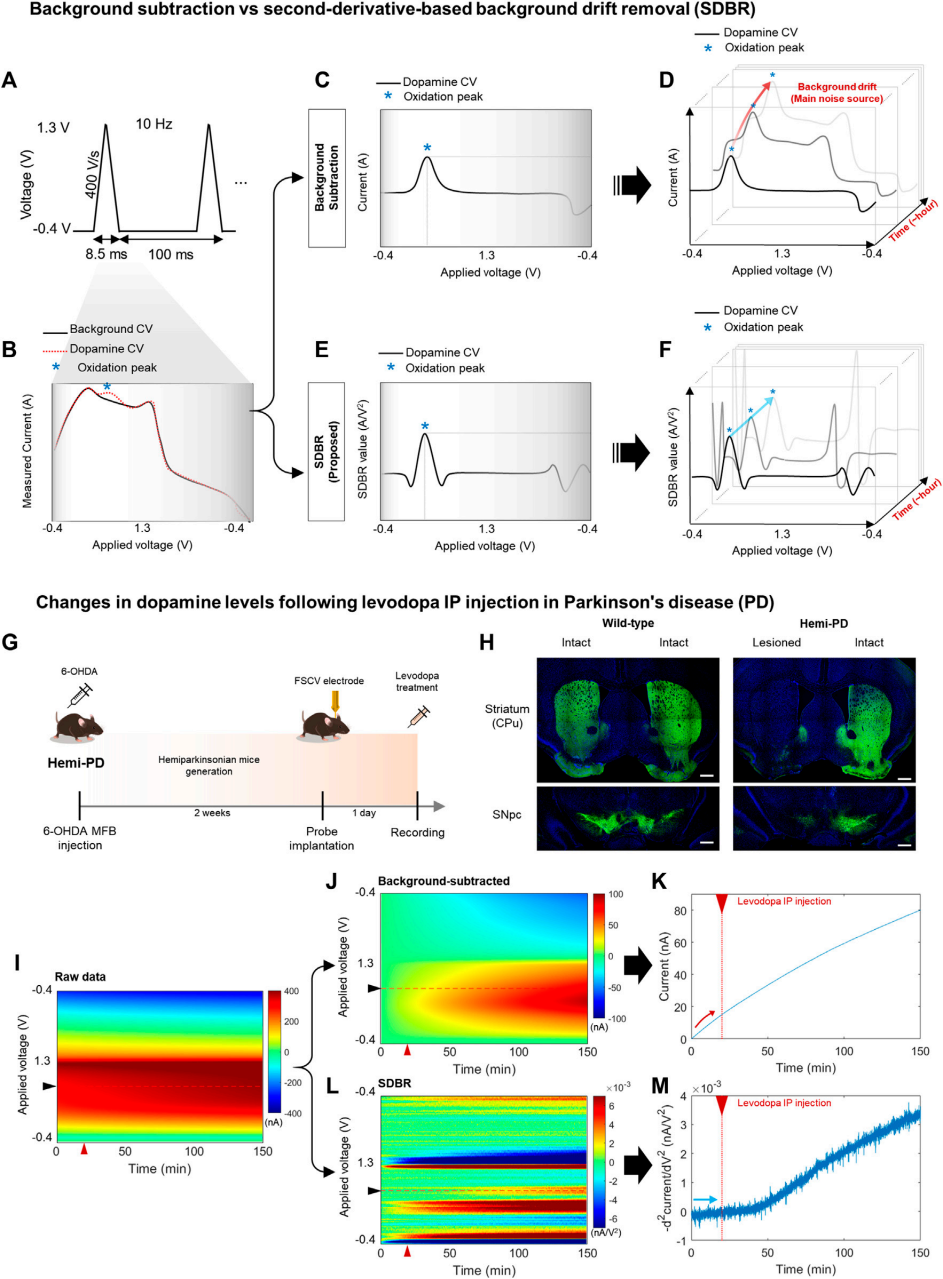
Long-range monitoring of tonic DA levels using an advanced FSCV system **(A–F)** Schematics of an advanced fast-scan cyclic voltammetry (FSCV) system compared with the standard method. **(A)** 10 Hz triangular voltammetric scans measure dopamine (DA) level changes using FSCV. **(B)** Background cyclic voltammogram (CV, black line), whose current is measured simultaneously against the voltage change, and DA CV (red dotted line), which indicates the DA oxidation peak (blue asterisk). **(C,D)** A conventional method that uses simple background subtraction is sufficient to estimate phasic DA changes based on the current amplitude of the oxidation peak within a short window. However, it is not ideal to trace tonic DA levels with repetitive scans over a longer window due to background drift in each scan. **(E,F)** The advanced method with second-derivative-based background removal (SDBR) measures the long-term dynamics of tonic DA levels simultaneously with its phasic change. This advanced method extracts tonic DA information by quantifying the curvature of oxidation peaks over repetitive scans, and thereby, it is not affected by background drift. **(G–M)** *In vivo* monitoring of tonic DA dynamics. **(G)** Schematics of FSCV application to the hemi-Parkinsonian (hemi-PD) model. **(H)** Histological validation of the hemi-PD mouse model. Unilateral lesion of dopaminergic neurons in the hemi-PD model compared with wild-type control mice. Scale bar: 500 µm. **(I–M)** Comparison between the conventional background subtraction method and the SDBR method to process raw FSCV data. Voltammogram data before and after levodopa treatment in the hemi-PD model. The red triangle marks levodopa treatment. **(I)** Raw data, **(J,K)** Post-processing using a conventional background-subtraction method, and **(L,M)** Post-processing using the SDBR method.

FSCV performs 10 Hz voltammetric measurements with CFME to detect changes to DA levels (Figure 1A). To observe relative changes in DA levels, FSCV compares cyclic voltammogram (CV) measures that follow changes to DA levels compared to baseline (Figure 1B). The difference in DA levels is identified using CV peaks around 0.6 V, which occurs due to the DA oxidation. A conventional background subtraction method effectively measures phasic DA signaling (Figure 1C). However, over several minutes and multiple scans, the DA peak current obtained using background subtraction slowly increases due to the background charging current (red arrow), which limits its ability to effectively trace tonic DA levels over time (Figure 1D). We processed FSCV data using the SDBR method, eliminating the background drift generated by charging current independent of DA concentration (Figure 1E). The advanced FSCV, alongside SDBR, extracts the tonic DA level by quantifying the curvature of the DA oxidation peak within each scan. Thus, the tonic DA level remains unaffected by background drift accumulation over repeated scans (blue arrow) (Figure 1F). In the previous study, we confirmed sensitivity and selectivity of the advanced FSCV technique with SDBR *in vitro* (Kang et al., 2022). Regarding the sensitivity of its method, we analyzed calibration plot against various dopamine concentrations (62.5–1,000 nM). The dopamine signals from our measurement display linear correlation with the dopamine concentrations tested *in vitro* and the lower limit of detection was 8.16 ± 0.08 nM, which is compatible with *in vivo* experiment. In addition, we further examined whether this measurement system distinguish dopamine from other similar molecules including levodopa (dopamine precursor), and 3,4-dihydroxyphenylacetic acid (DOPAC, dopamine metabolite) *in vitro*. In our measurement the selectivity of levodopa and DOPAC was below 1% of dopamine, indicating the high selectivity of the advanced FSCV with SDBR toward dopamine.

To examine whether this advanced FSCV method is effective for long-range measurements of tonic DA *in vivo*, we applied it to the pharmacodynamic analysis of levodopa (Figure 1G). To do this, we generated a hemi-Parkinsonian (hemi-PD) mouse model using a unilateral 6-OHDA lesion in the median fiber bundle (MFB). After 2 weeks of recovery, the animals were implanted with a CFME probe into the lesioned hemisphere and subjected to FSCV recording following acute levodopa administration. Immunohistochemical analysis of tyrosine hydroxylase, a dopaminergic neuron marker, verified clear degeneration of dopaminergic neurons and their axonal fibers at the striatum (caudate putamen: CPu) and substantia nigra pars compacta (SNpc). This was observed in the lesioned hemisphere of the hemi-PD model but not in the intact hemisphere of the model nor either hemisphere of wild-type (WT) mice (Figure 1H). In this hemi-PD model, we collected *in vivo* FSCV data for 150 min before and after levodopa injection. The voltammetric measurements from each 0.1 s scan were color-coded over time (Figure 1I). We have processed this raw data using two distinct methods: conventional background subtraction (Figure 1J) and SDBR (Figure 1L). The values of DA oxidation peaks measured in each scan were plotted over time. In the conventional background subtraction method, the current of DA oxidation peak voltage gradually increased over time, starting even before levodopa injection at 20 min and continuing throughout the entire recording (Figure 1K). This steady increase in the background charging current poses a challenge for the reliable measurements of DA dynamics over extended period. Contrarily, using the SDBR method, the DA oxidation peak value remained consistent before levodopa treatment, and it gradually increased only after the administration of the prodrug (Figure 1M). This pattern suggests the efficient elimination of background signal. These findings underscore the unique strength of this advanced FSCV system in enabling continuous, long-range measurement of tonic DA changes without systemic background noise interference. Collectively, our result suggests that the advanced combination of FSCV with the SDBR method holds promise for the pharmacodynamic analysis of levodopa in hemi-PD model mice.

### 3.2 Dyskinesia induction following chronic administration of levodopa in the PD model

**FIGURE 2:**
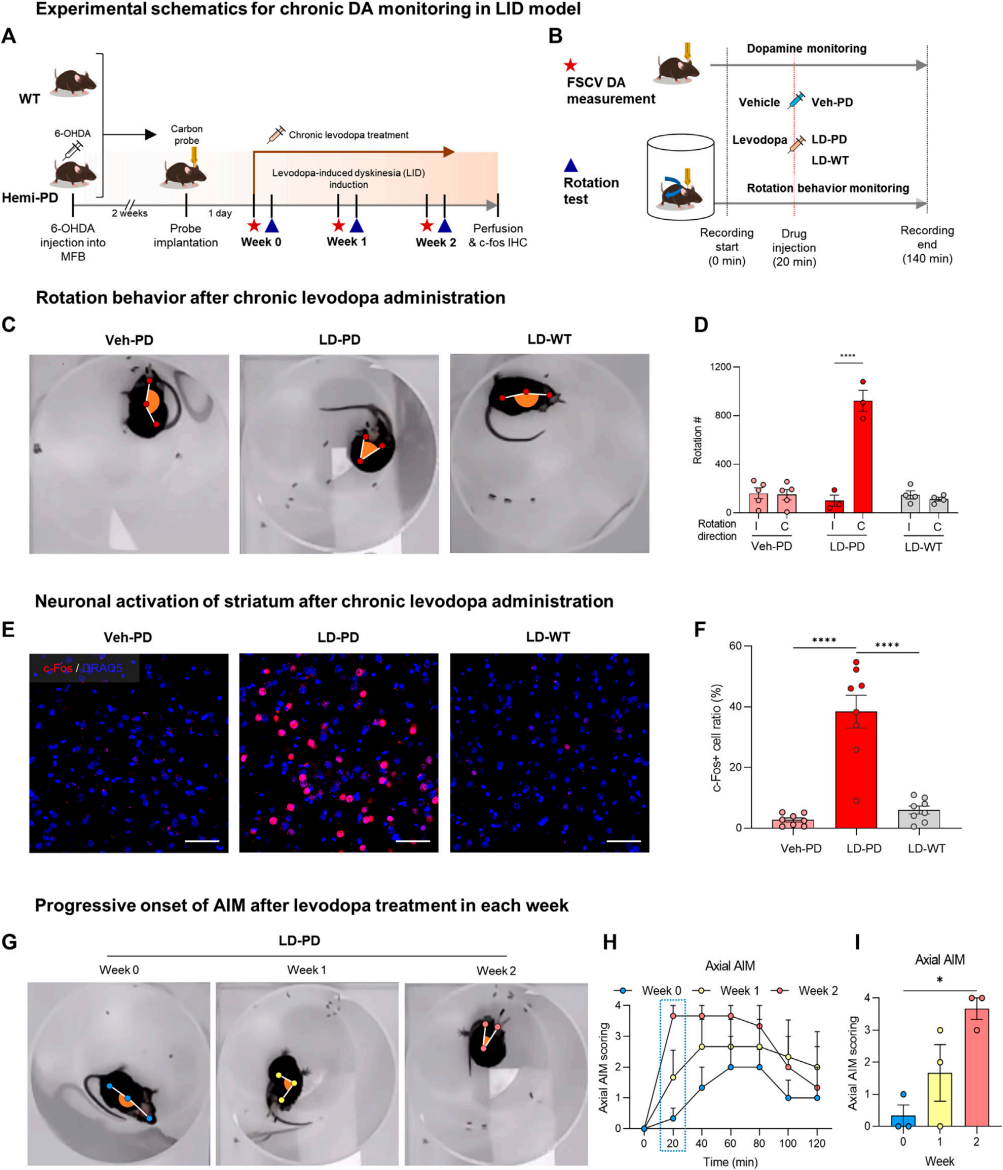
Animal model for levodopa-induced dyskinesia **(A)** Experimental schematics for chronic DA monitoring and behavioral testing following chronic levodopa treatment in the WT and hemi-PD models. All animals were implanted with carbon fiber microelectrode and subjected to daily levodopa treatment to induce dyskinesia for 2 weeks. During the progression of LID, FSCV recordings (red star) were collected, and rotation tests (blue triangle) were performed weekly. **(B)** Experimental timeline for FSCV recordings and rotation tests performed each week. The assay started at time 0 and ended at 140 min with either a vehicle or levodopa injection at 20 min, as indicated. **(C)** Top view of rotational behavior recordings after 2 weeks of levodopa treatment in each group of mice. **(D)** Clockwise rotation counts for Figure 2C. **(I)** Ipsilateral rotation, **(C)** Contralateral rotation. Differences within individual groups were statistically analyzed using a two-way ANOVA *post hoc* Šídák’s multiple comparisons tests (F_interaction_ (2, 18) = 47.24, df = 2, *p* < 0.0001; F_direction_ (1, 18) = 45.38, df = 1, *p* < 0.0001; F_mice group_ (2, 18) = 35.91, df = 2, *p* < 0.0001). Four asterisks (****) indicate *p* < 0.0001. **(E)** c-Fos immunohistochemistry for striatal neuron activation following drug treatment (c-Fos, red; DRAQ5, blue). Scale bar: 50 µm. **(F)** Quantitative analysis of c-Fos positive neurons vs. total striatal neurons in each group of mice. Differences within individual groups were statistically analyzed using an ordinary one-way ANOVA and *post hoc* Tukey’s multiple comparison tests (F_treatment_ (2, 21) = 37.05, df = 2, *p* < 0.0001). Four asterisks (****) indicate *p* < 0.0001. **(G)** The top view of rotational behavior test in hemi-PD mice after levodopa treatment each week. **(H)** Axial AIM scoring following levodopa treatment in the hemi-PD group. **(I)** Axial AIM scores were captured 20 min after levodopa treatment. Differences within individual groups were statistically analyzed using an ordinary one-way ANOVA test and *post hoc* Dunnett’s multiple comparisons test (F_treatment_ (2, 6) = 8.444, df = 2, *p* = 0.0180). Single asterisk (*) indicates *p* < 0.05.

Levodopa is metabolized into DA, which reverses hypodopaminergic conditions by ameliorating motor symptoms in patients with PD. However, chronic use of levodopa often leads to LID, abnormal involuntary movements immediately following acute levodopa administration. LID is successfully modeled in small animals with chronic levodopa administration after the degeneration of DA neurons (Sugiyama et al., 2021). In this study, the experimental paradigm was designed to examine whether tonic DA dynamics are altered with the onset of LID in the animal model (Figure 2A). Two weeks after the dopaminergic lesion, hemi-PD, and WT control mice were implanted with a CFME probe in the dorsal striatum. One day following the electrode implantation, all animals were administered daily levodopa (6 mg/kg) for 2 weeks. Advanced FSCV and behavioral tests were performed on two consecutive days at the beginning of each week (Figure 2B). To compare the effect of acute levodopa administration, the animals were further divided into three separate groups that were given either vehicle or levodopa, which were annotated as follows: vehicle-treated hemi-PD (Veh-PD), and levodopa-treated hemi-PD (LD-PD), levodopa-treated WT (LD-WT). Two weeks after chronic levodopa administration, LD-PD, but not LD-WT, started to display an apparent dyskinetic posture with a narrow head-to-body angle and a drastic increase in contraversive rotation following acute drug treatment (Figures 2C, D). Additionally, c-Fos immunohistochemistry revealed that acute levodopa administration induced hyperactivation of striatal medium spiny neurons (MSNs) in the lesioned side of the LD-PD group; however, this was not observed in the LD-WT group (Figures 2E, F) consistent with the onset of dyskinetic behavior in LD-PD. We further confirmed the progressive onset of dyskinetic behavior by scoring axial abnormal involuntary movement (AIM) each week (Figure 2G; Supplementary Movie S1) (Cenci and Lundblad, 2007). Axial AIM was triggered by acute levodopa administration to the LD-PD group only but not in other groups (Veh-PD, LD-WT); it was saturated around 20 min after administration and lasted approximately 1 hour (Figures 2H, I). In response to this acute drug administration, the axial AIM was exacerbated through chronic drug treatment over 2 weeks, suggesting progressive LID in this animal model. Taken together, these results demonstrate an established animal model to represent behavioral and histological features of LID onset in PD.

### 3.3 Progressive onset of dyskinetic behavior in a LID model

**FIGURE 3:**
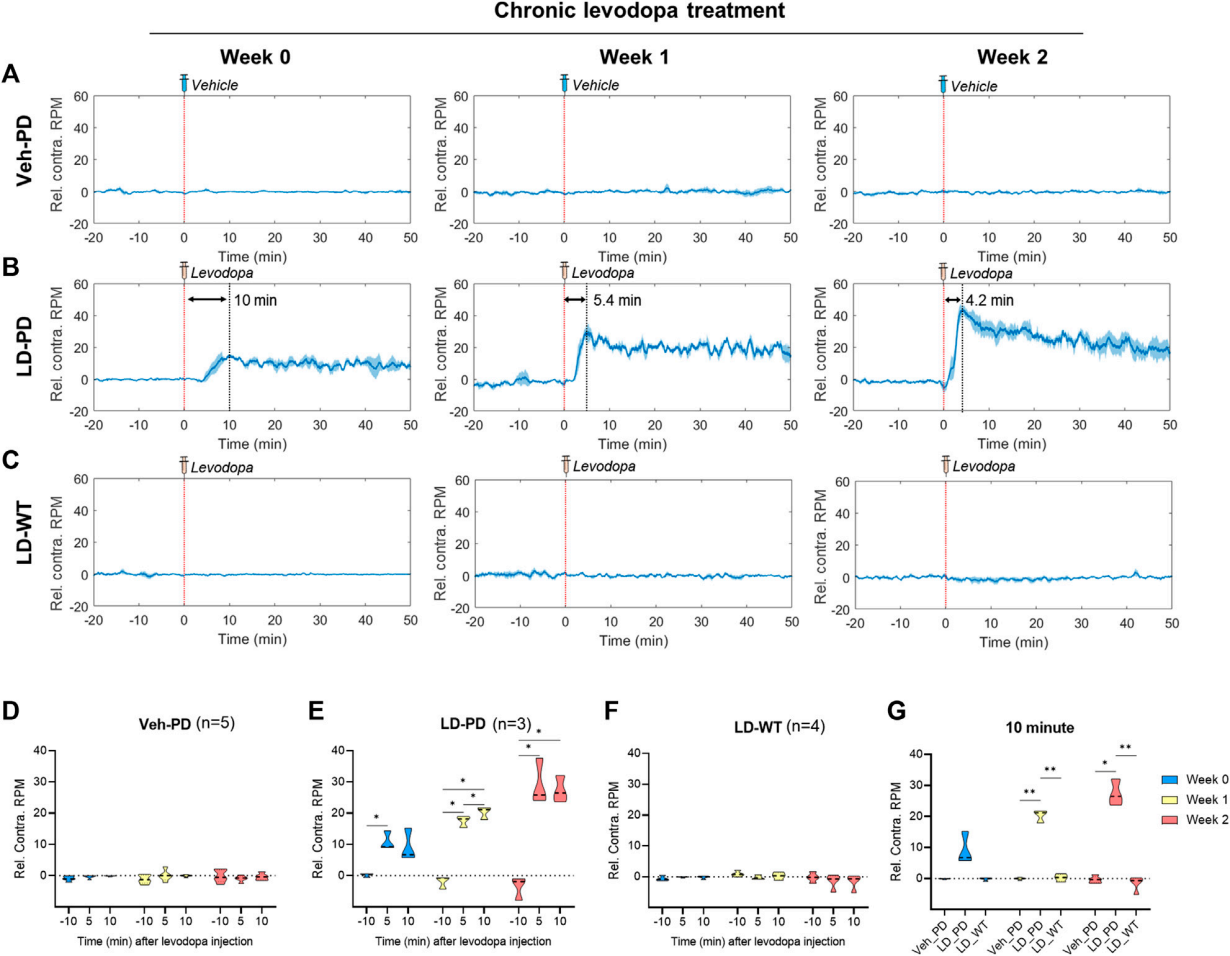
Profiling dyskinetic behavior in response to acute levodopa administration along LID progression **(A–C)** Changes to rotational behavior in response to acute administration of either vehicle or levodopa over 2 weeks (0, 1, and 2) in the **(A)** Hemi-PD vehicle group (n = 5), **(B)** Hemi-PD levodopa group (n = 3), and **(C)** WT levodopa group (n = 4). The red dotted line indicates time 0 for levodopa administration. The blue line shows the average values of individual data in each group over time. Blue shading represents the standard error of the mean for each dataset over time (Rel. Contra. RPM indicates relative contraversive rotation per minute). **(D–F)** The bar graph depicts the average and standard error of the mean DA level for −11 to -9-min (−10 min), 4–6 min (5 min), and 9–11 min (10 min) in the **(D)** Hemi-PD vehicle group, **(E)** Hemi-PD levodopa group and **(F)** WT levodopa group. **(G)** Mice group comparison at 10 min after drug injection. Differences within individual groups were statistically analyzed using a two-way ANOVA and *post hoc* Tukey’s multiple comparisons tests (Veh-PD: F_interaction_ (4, 24) = 1.622, df = 4, *p* = 0.2013/F_recording time_ (1.980, 23.76) = 1.333, df = 2, *p* = 0.2825/F_treatment period_ (2, 12) = 0.01314, df = 2, *p* = 0.9870; LD-PD group: F_interaction_ (4, 12) = 14.44, df = 4, *p* = 0.0002/F_recording time_ (1.355, 8.132) = 150.1, df = 2, *p* < 0.0001/F_treatment period_ (2, 6) = 9.787, df = 2, *p* = 0.0129; LD-WT group: F_interaction_ (4, 18) = 1.937, df = 4, *p* = 0.1479/F_recording time_ (1.636, 14.72) = 2.087, df = 2, *p* = 0.1641/F_treatment period_ (2, 9) = 1.267, df = 2, *p* = 0.3274; 10 min graph: F_interaction_ (4, 18) = 18.70, df = 4, *p* < 0.0001/F_mice group_ (2, 9) = 408.1, df = 2, *p* < 0.0001/F_treatment period_ (1.524, 13.72) = 16.72, df = 2, *p* = 0.0004). Single asterisk (*) indicates *p* < 0.05, and two asterisks (**) indicate *p* < 0.01.

Next, we further quantified dyskinetic behavior using high temporal resolution in the animal model to characterize the behavioral features of LID progression. Unilateral lesions of dopaminergic projection drive these hemi-PD model mice to exhibit mild ipsiversive rotation. However, acute levodopa treatment incites robust contraversive rotation. Here, we profiled time-dependent dyskinetic behavior in response to acute levodopa treatment throughout LID progression. We measured rotation counts each week in both ipsiversive and contraversive directions. All data from four comparison groups were used to calculate the difference in contraversive rotations per minute (RPM) and ipsiversive RPM, plotted over the time axis from −20–50 min following acute levodopa administration (Figures 3A–C). LD-WT mice group did not change in rotational behavior following acute levodopa treatment (Figure 3C), suggesting no sign of induced dyskinesia even after chronic drug administration over 2 weeks. In sharp contrast, hemi-PD mice (LD-PD vs. Veh-PD) exhibited robust contraversive rotation in response to acute levodopa treatment (Figures 3A, B). Interestingly, the peak times in contraversive rotation in LD-PD were week 0: 10.0; week 1: 5.4; and week 2: 4.2 min. The maximum RPM increased from week 0: 15.25; week 1: 30; and week 2: 43.67 RPM, in proportion to the duration of chronic levodopa administration, suggesting that acute levodopa-triggered dyskinetic behavior becomes increasingly severe with LID progression. In addition, we observed that LD-PD rotation behavior decreases gradually once it reaches its peak following acute levodopa administration. When we compared the contraversive RPM across the three groups before and after acute vehicle or levodopa administration, only LD-PD showed a drastic increase in contraversive rotation (Figures 3D–G). Collectively, these results indicate that LID progression accelerates dyskinetic behavior, likely due to a severe dopaminergic imbalance following acute drug administration.

### 3.4 Rapid induction of an excessive hyperdopaminergic condition with LID progression

**FIGURE 4:**
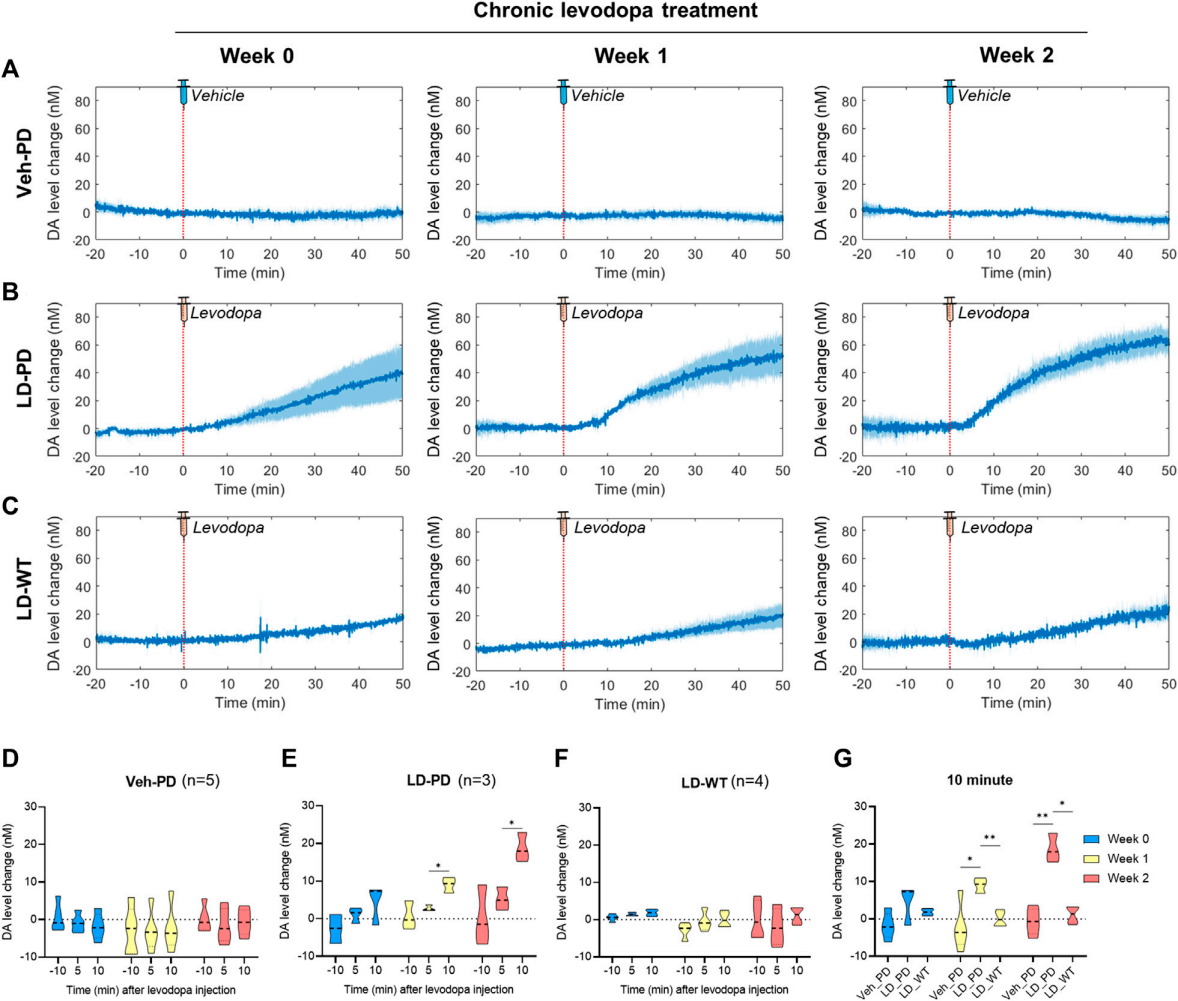
Long-range tracing of tonic DA dynamics in response to acute levodopa administration to the LID model using the advanced FSCV system **(A–C)** During chronic administration of levodopa over 2 weeks, long-range dynamics of tonic DA level were monitored weekly (0, 1, and 2) in the **(A)** PD vehicle group (n = 5), **(B)** PD levodopa group (n = 3), and **(C)** WT levodopa group (n = 4). The red dotted line marks time 0 for a single levodopa administration while FSCV recording is in progress. The blue line represents the average values of individual DA levels in each group over time. Blue shading represents the standard error of the mean for each dataset over time. **(D–F)** The bar graph depicts the average and standard error of the mean DA level for −11 to -9-min (−10 min), 4–6 min (5 min), and 9–11 min (10 min) in the **(D)** PD vehicle group, **(E)** PD levodopa group, and **(F)** WT levodopa group. **(G)** Mice group comparison at 10 min after drug injection. Differences within individual groups were statistically analyzed using a two-way ANOVA and *post hoc* Tukey’s multiple comparisons tests (Veh-PD: F_interaction_ (4, 24) = 0.5316, df = 4, *p* = 0.7137/F_recording time_ (1.128, 13.53) = 0.6707, df = 2, *p* = 0.4446/F_treatment period_ (2, 12) = 0.4096, df = 2, *p* = 0.6728; LD-PD group: F_interaction_ (4, 12) = 1.469, df = 4, *p* = 0.2719/F_recording time_ (1.060, 6.361) = 13.15, df = 2, *p* = 0.0095/F_treatment period_ (2, 6) = 19.18, df = 2, *p* = 0.0025; LD-WT group: F_interaction_ (4, 18) = 0.5758, df = 4, *p* = 0.6838/F_recording time_ (1.180, 10.62) = 1.038, df = 2, *p* = 0.3455/F_treatment period_ (2, 9) = 2.933, df = 2, *p* = 0.1045; 10 min graph: F_interaction_ (4, 18) = 5.814, df = 4, *p* = 0.0035/F_mice group_ (2, 9) = 20.35, df = 2, *p* = 0.0005/F_treatment period_ (1.949, 17.54) = 8.085, df = 2, *p* = 0.0034). Single asterisk (*) indicates *p* < 0.05, and two asterisks (**) indicate *p* < 0.01.

Here, we predicted abnormal changes to tonic DA levels in response to acute levodopa treatment in the LID model. We examined this hypothesis by analyzing levodopa pharmacodynamics in WT and hemi-PD models using the advanced FSCV system. Three groups (Veh-PD, LD-PD, LD-WT) were used to examine the effect of acute levodopa administration on tonic DA levels. Advanced FSCV was utilized to trace the continuous dynamics of tonic DA ranging from between 20 min before and 50 min following acute levodopa treatment. LD-WT mice displayed a slow to moderate increase in tonic DA levels (week 0: 19.26 ± 1.67; week 1: 23.25 ± 10.17; week 2: 24.87 ± 7.17 nM) after 50 min of levodopa injection (Figures 4C, F). In contrast, LD-PD mice displayed rapid and drastic surges in tonic DA levels in response to the same dose of levodopa (Figures 4B, E). Furthermore, we found that the maximum change in DA increased (week 0: 39.98 ± 19.05; week 1: 51.70 ± 15.66; week 2: 61.14 ± 7.61 nM), and its slope representing the speed of DA change became steeper along LID progression. In addition, the tonic DA level remained unaltered in the Veh-PD, reflecting the DA specificity of this advanced FSCV system (Figures 4A, D). When we compared the mean change in DA concentration for 10 min before and after acute vehicle or levodopa administration across the three groups, only LD-PD showed a rapid induction of excessive hyperdopaminergic condition (Figure 4G). Taken together, we demonstrated that chronic levodopa administration in the PD model induces abnormal transitions in levodopa pharmacodynamics alongside the gradual onset of LID.

### 3.5 Correlation between dyskinetic behavior and the tonic DA dynamics along LID progression

**FIGURE 5:**
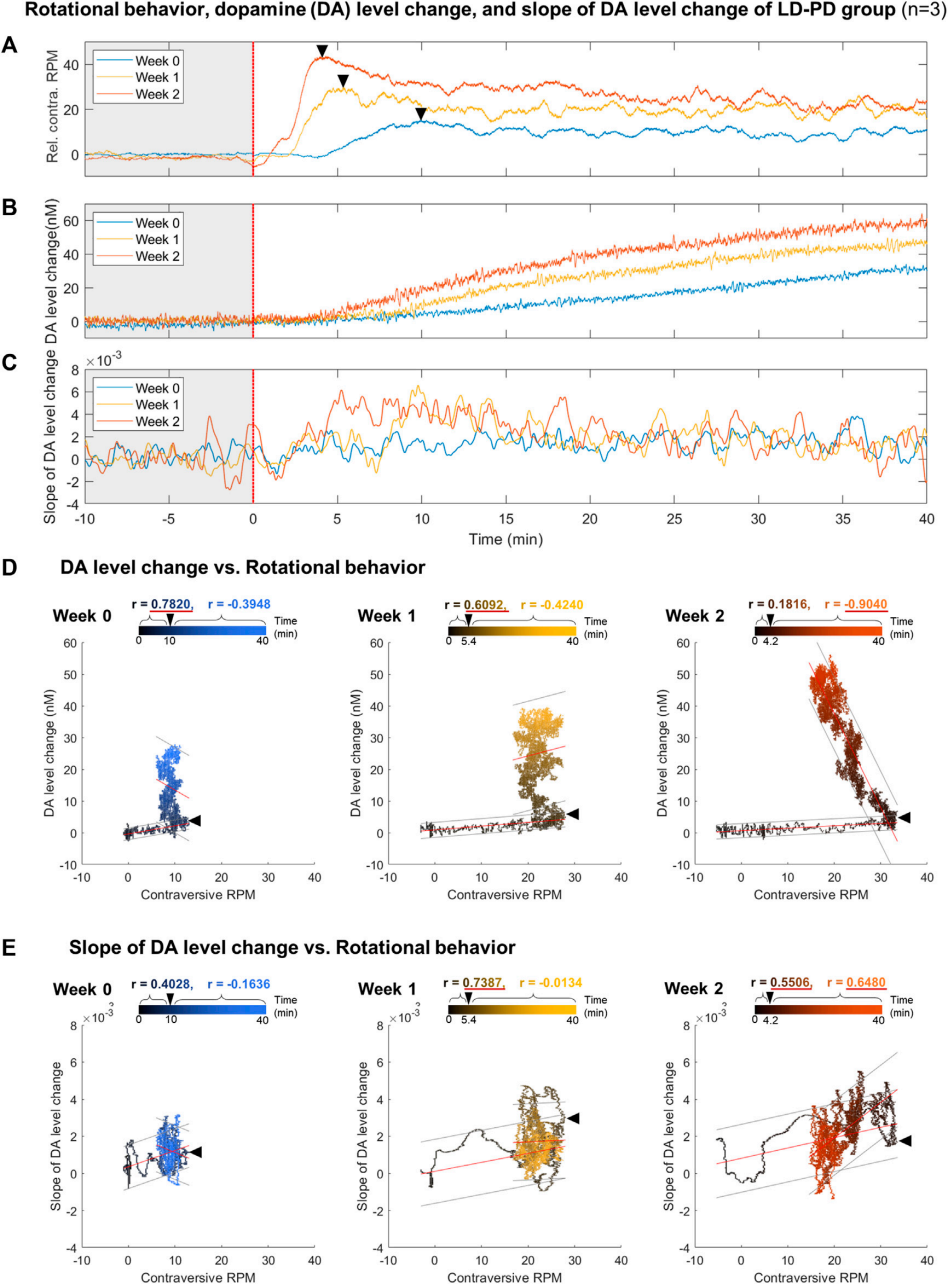
Correlation analysis between dyskinetic behavior and DA dynamics along LID induction **(A–C)** Comparison between **(A)** relative contraversive RPM of the rotational behavior, **(B)** DA level change, and **(C)** slope of DA level change in response to a single levodopa administration each week for the LID modeling procedure in PD levodopa group. All results were averaged with three LD-PD mice data (n = 3). The colors of individual lines, blue, yellow, and orange, represent weeks 0, 1, and 2, respectively. A black asterisk marks the transition time from when the rotation speed per minute was at the maximum value within 10 min of levodopa administration. **(D)** The scatter plot between the rotational behavior and DA level change. **(E)** The scatter plot between the rotational behavior and the slope of DA level change. Black and colored circles in the scatter plots represent data ranging from 0 to the end time point. Black arrowheads in the scatter plot mark the peak time points of rotational behavior. On the upper part of each scatter plot, the correlation coefficients (r) before and after the peak time points are depicted using black and colored text, respectively. If the absolute value of r is more significant than 0.5, it is underlined in red. All correlation coefficient calculations were statistically significant in a two-tailed *t*-test (*p* < 0.0001). The red lines above the circles indicate the fitted linear model and the gray lines indicate an estimated 95% prediction interval.

As outlined above, dyskinetic behavior and tonic DA level drastically increased in the LID animal model. Next, we investigated the correlation between these two factors across LID progression. The time-series data for rotational behavior (Figure 5A) and the change in DA level (Figure 5B) were matched by levodopa treatment at time 0 with three LD-PD mice data. Notably, the onset of rotational behavior occurred considerably earlier, even before the increase in tonic DA level became detectable in LD-PD. Furthermore, the tonic DA level continued to increase until the end of our assay after levodopa injection. In contrast, rotational behavior increased until the recorded peak (week 2: 4.2 min) and declined gradually despite a consistent increase in the tonic DA level in LD-PD. The apparent discrepancy between these two factors was further supported by examining their correlation. In week 2, we observed no correlation between these variables before the peak at 4.2 min (r = 0.1816) and a significant inverse correlation following the peak (r = −0.9040) (Figure 5D). Conversely, we explored the potential relationship between the change in DA levels over time and the severity of dyskinetic behavior through LID progression. The change in DA levels estimated the relative speed of DA increases each week (Supplementary Figure S1) and was plotted using the same period (Figure 5C). Remarkably, the slope of changing DA levels following acute levodopa treatment became steeper with LID progression. Furthermore, the slope at week two is significantly correlated with dyskinetic behavior following the peak time (r = 0.6480). In contrast, the slope at week 0 yielded no significant correlation before and after the peak time (r = 0.4028 and r = −0.1636, respectively). Our long-range measurement of levodopa pharmacodynamics demonstrates that the behavioral LID onset is strongly associated with changing tonic DA level dynamics, particularly reflected by the speed of excessive DA accumulation.

## 4 Discussion

Levodopa is a DA prodrug and is the most widely used medication in cases of PD. However, its long-term usage is often accompanied by LID, a detrimental side effect (Friedman, 1985). With the advanced FSCV technique combined with SDBR, we conducted long-range measurements of tonic DA dynamics with high temporal resolution in the rodent model of LID. We found that the behavioral onset of LID is strongly associated with the rapid induction of excessive hyperdopaminergic conditions in response to a single administration of levodopa. Here, we demonstrated that the advanced FSCV system is a valuable tool to address the molecular etiology underlying LID, particularly in the pharmacodynamic analysis of levodopa in PD.

DA serves as a critical neuromodulator governing voluntary movements, with its depletion causally linked to PD. DA levels in the striatum exhibit dynamic fluctuations across various timescales, including rapid transients lasting several seconds (e.g., phasic changes) and slow oscillations spanning over minutes to hours (e.g., tonic variations). Analyzing these dynamic shifts in DA enables a deeper understanding of DA functional role in a healthy brain and sheds light on PD pathology in both preclinical and clinical studies. Despite its significance, there remains a scarcity of *in vivo* techniques capable of effectively measuring tonic DA levels with high temporal resolution and across wide dose ranges over the long term. Fundamentally, the FSCV stands as an advantageous technique, allowing continuous, prolonged recordings and offering a broad detection range for DA levels (Venton and Cao, 2020). However, continuous FSCV amplifies noise signals, notably from chemical adsorption on the surface of the carbon probe, which poses challenges in signal detection. In the previous study, we developed an FSCV technique used in conjunction with the SDBR method and examined the DA selectivity and sensitivity of the technique *in vitro* and *in vivo* (Kang et al., 2022). In the current study, while building upon our prior findings, we have extended the application of this technique for *in vivo* pharmacodynamic analysis of levodopa-induced dyskinesia (LID) in the PD model. In the animal model of LID progression, this approach could extract tonic DA information from voltammograms generated every 0.1 s through second-order differentiation while effectively mitigating noise signals originating from the capacitive charging current. In the conventional background subtraction method, the current of DA oxidation peak voltage gradually increased over time, interfering the reliable measurements of DA dynamics over extended periods (Figure 1K). Conversely, using the SDBR method, the DA oxidation peak value remained unchanged, but began to increase only after levodopa administration (Figure 1M). These comparisons underscore the unique strength of this advanced FSCV with SDBR method in enabling continuous, long-range measurement of tonic DA changes *in vivo* without systemic background noise interference. Leveraging this advantage, we applied this technique to the animal model of the progressive onset of LID to profile the pharmacodynamic changes of tonic dopamine levels after a single levodopa administration (Figure 2). We observed the rapid surge in tonic DA changes from a depleted to a hyperdopaminergic state before and after a single levodopa treatment concurrent with LID progression. This approach revealed that the tonic DA increase following acute levodopa administration accelerated and became more excessive with the onset of LID (Figure 5). Therefore, we demonstrated that the advanced FSCV technique is compatible with the prolonged, continuous measurement of substantial fluctuations in tonic DA over the long term.

Long-term use of levodopa in PD patients leads to the development of dyskinetic behaviors, encompassing abnormal involuntary movements in arms, legs, body, and face (Friedman, 1985). In this study, we further investigated the correlation between tonic DA dynamics and the manifestation of dyskinetic behavior during the LID progression. To assess dyskinetic behaviors immediately after acute levodopa administration, we employed the rotational behavior, which is evident in hemi-PD model mice. Rotational behavior is a one of dyskinetic response that correlates with other rodent dyskinetic parameters in the hemi-PD model (Henry et al., 1998; Konitsiotis and Tsironis, 2006; Peng et al., 2019), resulting from DA imbalance between the left and right hemispheres. Contraversive rotation in the hemi-PD model reflects the severity of the dyskinetic response over time following levodopa administration. This response becomes more pronounced with the progressive onset of LID over 2 weeks of chronic levodopa administration (Figure 3). Concurrently, alongside behavioral testing, we continuously measured the tonic DA dynamics in the same animals using the advanced FSCV technique (Figure 4). In this model, we observed abnormal DA dynamics initiated and exacerbated with LID progression in the lesioned hemisphere of hemi-PD mice. With the high temporal resolution in both data sets (tonic DA dynamics and dyskinetic behavior) recorded within the same animals, we conducted a correlation analysis between these variables over time. Our data clearly demonstrated that the peak time associated with dyskinetic behavior was shortened, and its amplitude increased drastically with the progression of LID. These changes significantly correlated with the rapid induction of excessive hyperdopaminergic conditions within the same animals.

LID onset occurs through continuous levodopa treatment over time in PD patients (Friedman, 1985). Several studies describe the neurobiological mechanism underlying the behavioral onset of LID. Chronic DA depletion in the PD striatum gradually adapts to environmental variables. For instance, chronic levodopa administration in the PD induces hyper- and hypodopaminergic stress on MSNs and changes the DAR expression and sensitivity of MSNs (Gerfen, 2003; Aubert et al., 2005; Girasole et al., 2018). Notably, striatal MSNs change their protein expression and dendritic axonal arborization. Many animal model studies associate LID onset with post-synaptic abnormalities, including DA receptor 1 (D1) sensitization and direct MSN hyperactivity (Gerfen et al., 2002; Gerfen, 2003; Parker et al., 2018; Ryan et al., 2018; Sugiyama et al., 2021). D1 agonists and optogenetic manipulation of D1+ MSNs induce dyskinesia, whereas the pharmacological blockage of D1 suppresses LID, suggesting the essential function of D1+ MSNs hyperactivity that occurs with the onset of LID (Gerfen et al., 2002; Santini et al., 2009; Girasole et al., 2018; Parker et al., 2018; Ryan et al., 2018). Beyond the postsynaptic mechanisms described above, it is unclear whether the levodopa pharmacodynamics is altered with LID progression. By taking advantage of advanced FSCV, we successfully measured DA dynamics following a single dose of levodopa along the progressive onset of LID. Furthermore, we demonstrated its temporal correlation with dyskinetic behavior.

**FIGURE 6:**
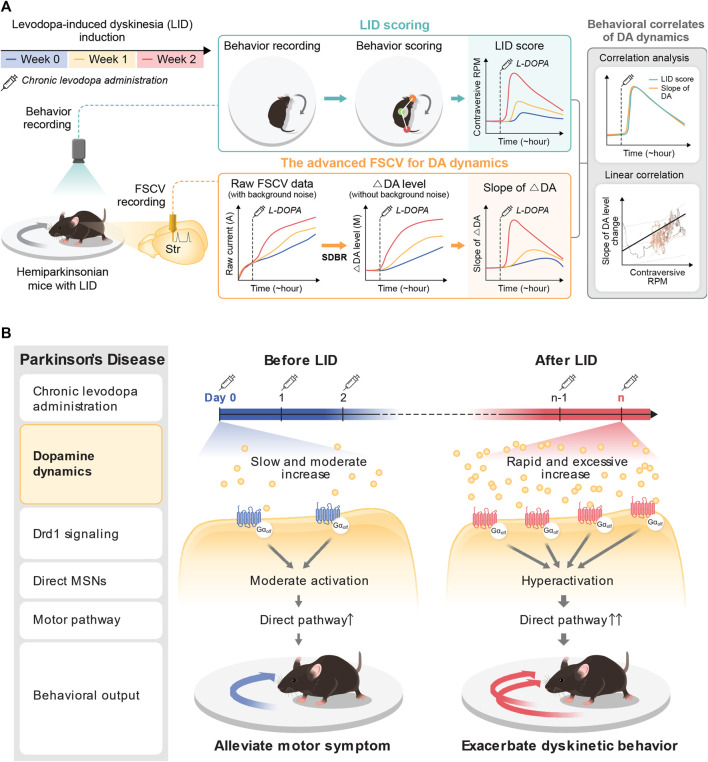
Pharmacodynamic analysis of LID with the advanced FSCV system. **(A)** Application of the advanced FSCV system to the LID model. Along with LID progression with chronic levodopa administration to the hemi-PD model, we traced the behavioral correlation of tonic DA dynamics after acute levodopa administration. The contraversive rotation, a dyskinetic behavior in the hemi-PD model, displays a solid correlation with the speed of DA increase, much less with the cumulative concentration of tonic DA. **(B)** A novel presynaptic mechanism underlying the pathological onset of LID. At the beginning of levodopa treatment in the hemi-PD model, the drug induces a slow and marginal increase of tonic DA level to alleviate the motor symptoms by resolving the hemi-PD model’s dopaminergic imbalance. With LID progression with chronic drug administration, acute levodopa administration provokes rapid induction of excessive hyperdopaminergic conditions. This novel presynaptic change of tonic DA level synergizes with hyper-sensitivity of Drd1+MSNs to exacerbate dyskinetic behavior with LID progression.

This study identified the accelerated accumulation of DA following acute levodopa administration alongside LID progression. The rapid induction of the hyperdopaminergic state reported in this study aligns with previous microdialysis research that reported excessive extracellular DA following levodopa treatment in 6-OHDA lesioned rats (Meissner et al., 2006). The initial levodopa administration to the hemi-PD model mice displayed a slow and moderate increase in tonic DA levels (39.98 ± 19.05 nM) without the noticeable emergence of dyskinetic response. In contrast, the same levodopa administration following LID causes a rapid surge of tonic DA alongside robust dyskinetic behavior (Figure 4). Furthermore, the cumulative concentration of tonic DA was substantially more significant, and the speed of DA change also became steeper along LID progression. These findings demonstrate that pulsatile levodopa administration results in abnormal swings of extracellular tonic DA with greater amplitudes observed alongside LID progression (Figure 5). Therefore, it is plausible that the abnormal fluctuation in tonic DA synergistically interacts with postsynaptic mechanisms and contributes to D1+ MSN hyperactivity and imbalance in motor pathways (Figure 6). Overall, we identified a presynaptic mechanism that altered the signature of tonic DA dynamics underlies the progressive onset of LID in PD.

In conclusion, we demonstrated that the advanced FSCV system is an invaluable tool for characterizing accurate long-range levodopa pharmacodynamics in the LID model. The behavioral onset of dyskinesia is associated with presynaptic abnormalities in tonic DA dynamics in LID. Although our understanding of LID molecular etiology is still rudimentary, the insights gained from this study elucidate fundamental LID mechanisms and present a critical step toward new diagnostic and therapeutic discovery using continuous DA monitoring for detecting the onset of LID.

## Data Availability

The raw data supporting the conclusion of this article will be made available by the authors, without undue reservation.
