# Supplementary material for: Continuous long-range measurement of tonic dopamine with advanced FSCV for pharmacodynamic analysis of levodopa-induced dyskinesia in Parkinson’s disease
Source: Front Bioeng Biotechnol. 2024 Jan 24;12:1335474. doi: 10.3389/fbioe.2024.1335474 (PMC10847580; doi:10.3389/fbioe.2024.1335474)
Supplement: Supplementary file 2 [file DataSheet1.docx]

Supplementary Material

# Supplementary Data


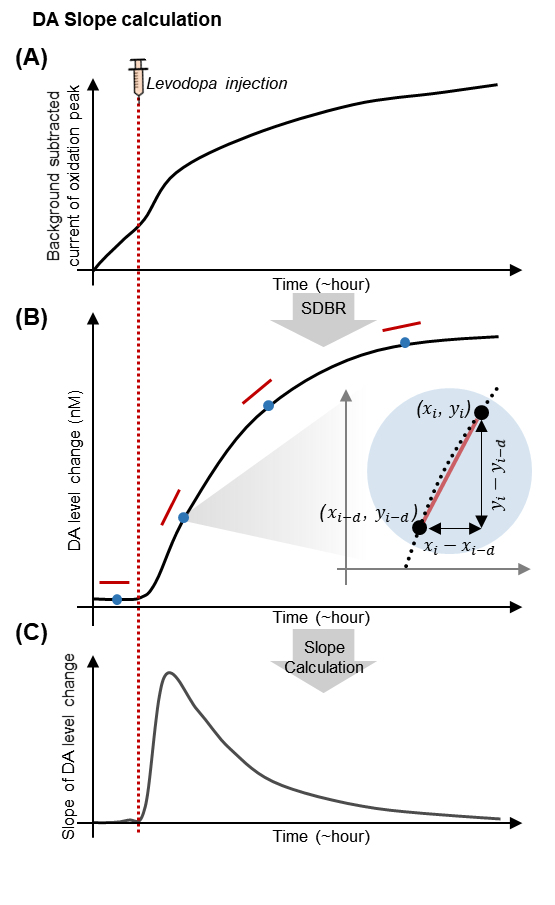


**Supplementary Figure 1.** Calculation procedure for the slope of DA level change.

(A) background subtracted current. (B) DA level changes. (C) slope of DA level changes following levodopa administration.


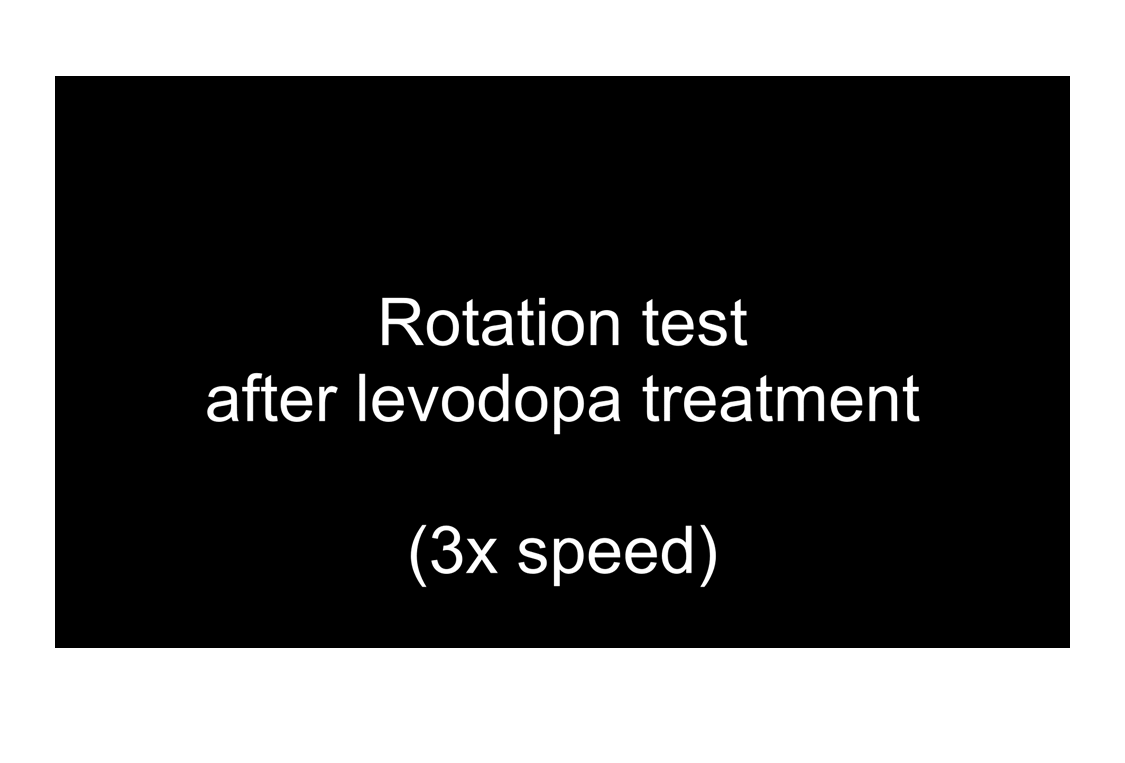


**Supplementary Movie 1.** Rotation behavior test following acute levodopa treatment
